# Factors influencing quality of life among cancer survivors in Kenya

**DOI:** 10.4314/ahs.v22i4.12

**Published:** 2022-12

**Authors:** Mary Kung'u, Lister Onsongo, James O Ogutu

**Affiliations:** 1 Community Health and Reproductive Health Nursing Department, School of Nursing, Kenyatta University, Nairobi, Kenya; 2 Pathology Department, School of Medicine, Kenyatta University, Nairobi, Kenya

**Keywords:** Quality of Life (QoL), Kenya, cancer patients

## Abstract

**Background:**

The number of cancer survivors is growing continuously due to advances in treatment of cancer patients. In developed countries, numerous studies on quality of life (QoL) of cancer survivors have been conducted. Little is known regarding the QoL of cancer survivors in Kenya. Therefore, the aim of this study was to explore the factors that contribute to QoL of cancer survivors in Kenya.

**Methods:**

A descriptive cross-sectional design was used for the study. Participants were 108 adult cancer survivors. Self and interviewer administered QoL Patient/Cancer Survivor Questionnaire was used

**Results:**

Findings indicate a statistically significant relationship between QoL and age (rs =-.055 p<.05), marital status (rs=.490 p<.01), income (rs =.228 p<.05), stage of cancer diagnosis (rs =-.269. p<.01), year of cancer diagnosis (rs =-.295 p<.01), religious affiliation (rs =-.279, p<.01) and the religion one belonged to (rs =-.198 p<.05). Regression analysis showed that age, stage of cancer diagnosis, time off treatment, educational level, and religious affiliation are significant predictors of QoL in cancer survivors.

**Conclusions:**

The findings highlight the importance of key factors associated to QoL in cancer survivors in Kenya. Interventions aimed at early cancer detection, treatment, and spiritual support among cancer survivors will improve QoL

## Introduction

Cancer is a serious problem in all populations regardless of age, sex, race, and socioeconomic status. Globally, it is the second leading cause of death and responsible for an estimated 9.6 million deaths 15. It is estimated that in 2020 the number of new cancer cases each year will reach 15 million. The predicted global cancer burden by 2040 is expected to exceed 27 million new cancer cases per year, a 50% increase on the estimated 18.1 million cancer cases in 2018, with the greatest increase in low income countries 15. It is also seen that low-income countries contribute to a majority of the 50% increase in the cancer cases. In sub-Saharan Africa, the cancer incidence burden is likely to be over 85% and according to 2018 data, and the estimated cancer deaths were around 506,000^15^.There is high likelihood of survival among cancer survivors as most of the incurable cancers have turned to be curable due to advances in biomedical re- search and cancer treatment 3.

In Kenya, cancer is the third leading cause of mortality after infectious and cardiovascular diseases and second among non-communicable diseases 15. Although population-based data does not exist, cancer incidence is estimated to be about 28000 cases, and the mortality to be over 22000.Over 60% of those affected are below the age of 70 years. The five most common cancers in Kenya include; breast, cervical, esophageal, prostate and colorectal cancer 15. Cancer survivors' QoL is a distinct element in determination of their survivorship. The world health organization defines QoL as a person's understanding of their place in life in relation to culture and value systems and this involves the person's goals, standards, expectations and concerns. QOL revolves around factors like the patient's health status, beliefs, social relations, environment and psychological status. It has been identified to provide prognostic information on prediction of cancer patients survival duration for various cancers 11. It is a multidimensional construct with a range of domains including; the physical, functional, psychological, economic, social and spiritual domain 1. QOL of cancer patients can be positively or negatively affected. For any cancer patient, their QoL is an important concern from the time of diagnosis and throughout the treatment. It is recognized as a measure for providing optimum management and care in oncology practice since it reflects the patient's impression of the influence cancer has during its diagnosis and treatment 17. Assessment of the QoL of cancer patients could lead to delivery of better care services since health care workers are able to assess the areas that they need to improve on. It also acts as an independent predictive factor along with the medical frameworks that are used in the treatment of breast cancer.

Sociodemographic factors affecting QoL include; age, marital status, gender, level of education and employment status 3. Age was identified to be a significant predictor for QoL. Young people portrayed lower social functioning compared to older persons while women rated their social relation on QoL to be higher than that of men 1. This was attributed to the high social interaction and participation of women than that of men. Can et al., study^6^ indicated that there is no association between marital status and QoL but only the social support may have contributed to the married people having better QoL than single ones.

Patients with breast cancer have the worst social outcomes hence poor QoL compared to patients with head-neck, sarcoma and gynecological cancers 6.Young breast cancer survivors showed significantly worse QoL outcomes compared with those diagnosed with cancer at a later age group. The younger individuals experienced higher levels of social inhibition while undergoing treatment as compared to the older age group 20. Patients with high duration of illness were associated with better QoL 18. Also, cancer patients on radiother- apy portray poor QoL than those on chemotherapy or post-surgery. This is because radiotherapy is more painful than chemotherapy and surgery and also has an advanced perceived stress 18.Individuals with high religious affiliation and practices in the United States were noted to have an improved QoL compared to those who did not have any religious affiliation 14.

Cancer survivors have identified spirituality as a way of coping with the cancer diagnosis. This tends to boost their spiritual wellbeing hence improving their QoL 20.

Various studies including 1, 14 and 18 identified various sociodemographic factors, and disease characteristics such as stage of cancer diagnosis and treatment modalities to have a significant association with QoL. There is a paucity of information regarding the QoL of cancer patients in Kenya, therefore the purpose of this study was to assess factors influencing QoL among cancer patients in Kenya.

## Methods Study design

A descriptive cross-sectional research design was used to investigate the association between QoL of cancer survivors with their sociodemographic, disease characteristics, and spiritual factors. Self and interviewer administered questionnaires were administered. Ethical approvals from the university, the hospital, and National Commission for Science, Technology, and Innovation were obtained.

### Study population

The study involved 108 cancer patients aged between 21–86 years with the cancer diagnosis for more than six months selected through systematic random sampling. The researcher went through the medical records of the patients due for review in the facility to identify the patients eligible for the study. On arrival to the clinic, every 2nd patient was introduced to the study and explained what the study was about including the benefits and the risk of participating. The patients had visited the hospital for follow up post treatment and had not expe- rience recurrence of cancer nor restarted on any cancer treatment.

### Measures

Information on sociodemographic, disease characteristics, spiritual factors and QoL was collected using a standard questionnaire with three parts.

### Sociodemographic variables

The first part included demographic variables such as age, sex, marital status, educational level, income, and diagnoses.

### Quality of life

The second part of the questionnaire was the Quality of Life Patient/Cancer Survivor Version (QOL-CSV) which comprised of 41 items based on the Likert scale of 0–10. The tool is divided into physical, psychological and spiritual subtopic each having related questions. Score of 0 was considered as worst outcome and 10 best outcomes. Several items had a reverse anchor and hence when coded needed to reverse the score of those items. The higher the score the patient obtained, the better the QoL he or she had. The scoring was based on the QOL-CSV scoring manual.

### Statistical Analysis

Data was analyzed using SPSS version 25.0. The demographic and disease-related characteristics of the participants were analyzed using frequency, percentages, means, and standard deviations. Spearman's rho was conducted to determine the relationship between demographic, disease characteristics, spiritual variable and QoL. Stepwise Multiple regression was conducted to predict overall QoL.

## Results

From the sociodemographic data of participants, the mean age of patients was 51±15.9 years with a range of 21–86 years and an overall QoL mean of 183.81±45.14. Majority of the respondents were female (52.8%) and, 41 (38%) of them had their education up to secondary level. Majority, 49(45.4%) were married, 39(36.1%) were employed and earning a salary. Most of those with income, about 33(30.6%) had an income of (ksh.30000-ksh.60000) $300 to $ 600 with less than 5% earning above $ 600(ksh.60000). (Table 1)

**Table 1:** Participants characteristics ( =108)

Variables	Categories	M ± SD	*(%)*
**Age**		50.64±15.9	
**PhWB**		46.24±14.31	
**PsWB**		59.47±29.47	
**SoWB**		38.93±17.24	
**SpWB**		39.35±17.78	
**Overall QOL**		183.81±45.14	
**Gender**	Male		51(47.2)
	Female		57(52.8)
**Marital status**	Single		17(15.7)
	Married		49 (45.4)
	Separated		11(11.2)
	Widowed		31(28.7)
**Education level**	Non-basic		17(15.7)
	Primary		18(16.7)
	Secondary		41(38.0)
	College		21(19.4)
	University		11(10.2)
**Employment status**	Employed		39(36.1)
	Self-employed		34(31.5)
	Not working		35(32.4)
**Income**	No income		35(32.4)
	Below ksh.10,000		11(10.2)
	ksh.10,001–30,000		24(22.2)
	ksh.30,001–60,000		33(30.6)
	ksh.60,001–100,000		5(4.6)
**Cancer stage**	I		4(3.7)
	II		50(46.3)
	III		25(23.1)
**Treatment**	CTx		18(16.7)
	CTx and surgery		48(44.4)
	RT and CTx		28(25.9)
	RT,CTx and surgery		14(13.0)
**Religious affiliation**	Yes		94(87)
	No		14(13)
**Religion**	Christians		79(84.0)
	Muslims		13(13.9)
	None		2(2.1)
**Contented with faith** **in God**	Extremely contented		36(33.3)
	Very much contented		29(26.9)
	Moderately contented		14(13.0)
	A little contented		15(13.9)
	Not at all contented		14(13.0)

**Key:** CTx, Chemotherapy; RT, Radiotherapy, N=frequency, %-percentage, SD=Standard deviation PhWB –Physical Wellbeing, SoWB –Social wellbeing, PsWB-Psychological Wellbeing, SpWB-Spiritual Wellbeing.

Majority of the participants 50 (46.3%) had stage II cancer, 48(44.4%) had received chemotherapy and surgery combination therapy. Majority of the participants 94 (87%) had a religious affiliation, 79 (84%) were Christians. Most 36 (33.3%) of the participants were extremely con- tented with faith in God and 28(25.9%) minimally participated in religious activities. This is as shown on Table 1.

There was a statistically significant correlation in relation to the variables of age, gender, education level, marital status and income. Younger individuals portrayed to have better PhWB, PsWB and overall QoL compared to the older participants (rs= 223, p<.05, rs = 248 p<.05, rs = 055 p<.05). In terms of gender, women experienced worse PhWB compared to men (rs = 253, p<0.01). Married participants had better PsWB, SoWB, SpWB and overall QoL compared to single partic- ipants (rs =.406 p<.01, rs =.500 p<.01, rs =.210 p<.05 rs=.490 p<.01). Participants with high income had a better PsWB, SoWB and overall QoL (rs =.271 p<.01, rs= .246 p<.05, rs =.228 p<.05). (Table 2)

**Table 2:** Correlation of socio demographic, disease and spiritual variables and QoL (n=108)

Variable	*QOL*	*PhWB*	*PsWB*	*SoWB*	*SpWB*
Age	-.055*	-.223*	-.248*	-.040	072
Gender	-.167	-.253**	.072	.062	.107
Education	.189	.219*	.226*	.234*	-.065
Marital status	.490**	-.216*	.406**	.500**	.210*
Monthly income	.228*	.183	.271**	.246*	.110
Treatment cost	-.070	.042	-.143	.037	-0.11
Cancer type	.091	-.110	-.062	.111	.123
Cancer stage	-.269**	-.498**	-.320**	.342*	.207*
Years with cancer dx	-.295**	-.116	-.118	.241*	.337**
Treatment used	.011	-.125	.162	-.035	.002
Complentary therapy	-.081	-.015	-.142	-.028	.074
Memberofreligious affiliation	-.279**	.169	-.151	-.230*	-.346**
The religion you belong to	-.198*	.125	-.103	-.131	-.295**
Spiritual support	.141	.013	.002	.075	.290**
Contented with faith in God	-.215*	.037	.178	.179	-263**
Spiritual life change	-.091	.074	-.087	.001	-.149
Source of social support	.155	-.129	.174	.102	.110

Key: * *p* < .05 ** *p* < .01 *** *p* < .001.

Patients with stage one cancer had better PhWB and overall QoL compared to patient with advance cancer (rs = 498. p<.01, rs= 269. p<.01). Individuals with advanced cancer had better PsWB and SpWB compared to individuals with stage one cancer (rs =.342 p<.05, rs=.207 p<.05). Individuals with recent diagnosis had bet- ter QoL compared to individuals with long term diagno- sis (rs = 295 p<.01). Subsequently, those who were diag- nosed earlier had better PsWB and SpWB compared to those with recent diagnosis (rs = 241 p<.05, rs=.337 p<.01). Participants with a religious affiliation had a bet- ter overall QoL, SoWB and SpWB compared to partici- pant with no religious affiliation (rs= 279, p<.01, rs = 230 p<.05, rs =.346 p<.01). Christians had better overall QoL and better SpWB compared to Muslim participants and those who did not belong to any religion (rs = 198 p<.05, rs = 295 p< .01). Individuals who were extremely contented with God had better QoL and SpWB compared to those who were not at all content- ed (rs = 215 p< .05, rs = 263 p<.01). This is as shown on Table 2.

**Table 3:** Predictors of overall QoL

*Variables*	*B*	*β*	*Df1*	*Df2*	*Adj.* *R^2^*	*T*	*p*	*F*
(Constant)	57.166		*5*	102	.500	3.025	.003	22.411
Age	-11.972	-.502				-6.990	.000	
Stage of cancer dx	-9.062	-.233				-3.379	.001	
Time off treatment	9.169	.217				3.164	.002	
Educational level	9.078	.195				2.739	.007	
Religious affiliation	3.297	.173				2.523	.013	

**Key:** Sig p < .05 while CI 95%, CI: Confidence Interval, β: Beta coefficient, t: t value while B coefficient is significant, Adj. R^2^: Adjusted coefficient of determination, dx; diagnosis.

Using variables that were significantly correlated with QoL, stepwise regression predicting overall QoL was done.50% of variance in the Qol was accounted by five predictors collectively (F (5,102) =22.41, P<.001). Looking at individual contribution of the predictors, the results show that time off cancer treatment (β=.217, t=3.164, p=.002), educational level (β=.195, t=2.739, p=.007) and religious affiliation (β=.173, t=2.523, p=.013) positively predict QoL. The results also show that young individuals with longer time off treatment, has attained tertiary education and have religious affiliation are more likely to report better Qol (β=.502, t= 6.990, p<.001). This is as shown on Table 3.

## Discussion

The study demonstrated a statistically significant relationship between sociodemographic variables and QoL in relation to the variables of age, gender, education level, marital status and income. Younger individuals had better QoL but worse PsWB compared to the older patients. These results were similar to those of a study by 1 where breast cancer survivors diagnosed at older age had worse PhWB and the general QoL. However, findings by 2 had contrasting results whereby older individuals had better QoL. This may be due to the ability of the young to cope with the cancer signs, symptoms and the side effects of cancer treatment. The results by 2 may be due to inclusion of a high number of older persons in the study. The results also showed that gender did not have a significant association with QoL. The results were similar to a study done among patients with brain tumor which showed gender not having a significant as- association with QoL^5^. This was contrary to studies in India 8 where women had better SoWB due to their high interaction and social support. The results may be due to variety of symptoms experienced by the cancer patients affecting both men and women.

Marital status had a significant association with overall QoL where patients who were single had worse QoL, PsWB, SoWB and SpWB compared to the married participants. Previous studies indicate married patients to have better QoL, PsWB, SoWB and SpWB 5. This is contrary to studies on marital QoL 9 where marital status did not have any influence on the QoL and only individuals with good social support had better QoL. The results may be attributed by the high social interaction and support portrayed by married people. Education had a significant correlation with QoL where individuals with tertiary education portrayed better PsWB, SoWB, SpWB and overall QoL compared with individuals with non-basic education. This was similar to 6 studies. The results were contrary to Ran et al., 17 study where level of education did not affect the QoL of the patients. Patients who are more educated have better understanding of the disease hence able to implement measures to improve their QoL. Income had a significant correlation with QoL. Participants with high income had better PsWB, SoWB and overall QoL. This was similar to a study done in Turkey among cancer patients where house- wives portrayed worse SoWB and QoL than other occupations 6. Lower QoL among those not working might be associated with them being isolated from social life and having less social and economic support.

The results show a statistically significant correlation between QoL domains with stage of cancer diagnosis and years with cancer diagnosis. Patients with stage one cancer had better PhWB, SoWB and QoL compared to patient with advance cancer. The results were similar to those of a study done among oropharyngeal cancer patients 17. This may be attributed by worse symptoms portrayed by patients with advance cancer and also the several combinations of treatments given to them. The results also show that cancer patients recently diagnosed with cancer had better QoL compared with individuals with long term diagnosis. This was similar to Cigno study 7 where cancer patients with long term diagnosis portrayed poor QoL. The results were contrary to study 18, which found that high QoL was associated with patients with long duration on illness. Their argument was that long term diagnosed patients may have been successfully treated or may have learnt how to manage some of the social and psychological problems associated with cancer diagnosis leading to better QoL. The results may be due to the negative outcomes of cancer on all QoL domains, the several therapies given and also the progression of the disease.

The findings found no statistical significance correlation between QoL domains with the type of cancer the patients had and the complementary therapy used (P>.05). These results were contrary to those of 18 where breast cancer patients had higher QoL compared with those with all other reproductive system cancers. The results may be due to inclusion of all types of cancers in the study and not classifying them in any major groups. The study found significant differences when comparing patients on different subgroups in terms of the treatment modality used where patient on chemo- therapy and surgery combination therapy had better QoL while radiotherapy patients portrayed worse QoL. The results were similar those of a study by 19 and 18. The poor QoL portrayed by patients on radiotherapy may be due to the many side effects of radiotherapy to the patients.

The findings of the study revealed a statistically significant relationship of QoL domains in relation to religious affiliation, the religion the patients belong to and how contented with faith in God one was. Participants with religious affiliation had a better a QoL, SoWB and SpWB compared to participant with no religious affiliation. Religious affiliations was in relation to having faith in a spiritual being and not necessarily belonging to a particular religion or doctrine. The findings of the study were similar to those of a study done in the United States of America among cancer survivors where spiritual beliefs remained relevant even to those who did not profess any religious faith 14.

The results also showed that Christians had better QoL and better SpWB compared to Muslim participants and those who did not belong to any religion. The results were contrary to 10 and 13, which showed being a Muslim was associated with better SpWB subscale. Islamic faith and beliefs contributed greatly to the overall spiritual wellbeing and having any psychological problem is a test to one's faith 17. The difference in the results may have been contributed by the low number of Muslim patients included in the study as participants. The results also show that individuals who were extremely contented with God and were active participants in religious activities had better SpWB compared to those who were not at all contented. This was similar to a study by Cigno where cancer patients who portrayed hopefulness and contentment in God equivalently had better QoL 7. Involvement in religious activity contributed greatly to improving spirituality hence helped them cope with the cancer disease resulting to better QoL.

The study finding shows that overall QoL was predicted by five variables including; age, education level, religious affiliation, stage of cancer diagnosis and time off cancer treatment. Education level, time off cancer treatment and religious affiliation positively predict QoL while age and year of cancer diagnosis negatively predicted QoL. According to 12 longitudenal study, only self-rated health and age predicted QoL. According to 4 cross-sectional study, positive social interactions was the overall predictor of QoL and age did not at all predict QoL. The difference noted may be attributed to differences in measures of QoL used and the type of study conducted.

The study indicate the relationship between age, gender, education level, marital status, income, stage of cancer diagnosis, time off cancer treatment, religious affiliation and QoL. Health care providers should not only focus on the kind of treatment the patients is on but also on other socioeconomic and disease related factors which have a great implication on the QoL of the cancer patients. The finding of the study can be utilized in development of programs that focus on the QoL of the cancer patients. The limitations of the results is the study being cross-sectional in nature hence does not establish relations of cause and effects making the study restricted to one centre. The study did not focus on the possibilities of other comorbidities on the cancer patients that could possibly influence their QoL. Future research should focus on effects of comorbidities on cancer patients QoL.

## Conclusion

The study highlights the key sociodemographic factors, disease characteristics and spiritual factors associated with QoL of cancer survivors in Kenya. The study demonstrates financial, psychological and spiritual support sig- nificantly improving the QoL of the cancer patients. The results on relationship between disease characteristics and QoL supports the clinical findings and may help in de- signing more specific strategies to help improve cancer patients QoL. The findings suggest need for interventions aimed at early cancer detection, treatment, and spiritual support among cancer survivors which will improve QoL.
